# International Hahnemannian Association: Eleventh Annual Meeting

**Published:** 1890-08

**Authors:** 


### INTERNATIONAL HAHNEMANNIAN ASSOCIATION.—ELEVENTH ANNUAL MEETING.

WATCH HILL, RHODE ISLAND, June 24th–27th, 1890.

### SESSION OF JUNE 24TH.

*Morning.*—The President called the meeting to order at 11.15 A. M.

The President then read his opening address.

On motion of Dr. Rushmore, a committee was appointed upon the President's address.

The committee was composed of Drs. Rushmore, Wesselhœft, and Butler.

A programme for the order of business was then adopted.

The report of the Secretary was received and referred to the Auditing Committee.

The report of the Treasurer was received and referred to the Auditing Committee.

The Auditing Committee was composed of Drs. Rushmore, Custis, and Powel.

Amendment to the by-laws, by Dr. Clark, was withdrawn.

Reports of delegates were received and referred to the Publication Committee. Reports were received from Drs. Rushmore, Sawyer, Powel, and Hitchcock.

Dr. Rushmore called the attention of the Association to the prolonged illness of the venerable Dr. P. P. Wells, of Brooklyn.

Dr. Fincke read a letter from Dr. Wells.

Dr. Butler also drew attention to the illness of Dr. E. A. Ballard, of Chicago, who is held in high esteem by all Hahnemannians.

A committee was appointed to send telegrams of respect and sympathy to Drs. Wells * and Ballard. The committee was composed of Drs. Butler, Wesselhœft, and Rushmore.

*Afternoon.*—Dr. B. L. B. Baylies, of Brooklyn, was appointed temporary chairman of Bureau of Obstetrics.

The Bureau of Homœopathics was then made the order of the session. Dr. C. W. Butler, chairman.

A paper by Dr. Lowe was read by Mrs. Butler.

A paper by Dr. T. P. Wilson was read by the chairman. A paper by the chairman himself was also read, and it was followed by a discussion.

Dr. Fincke read a paper upon Sections 59 to 66 of *The Organon.* After discussion, the meeting adjourned to 8 P. M.

*Evening.*—The order of the afternoon session was resumed.

Dr. James T. Kent read a paper upon Sections 63, 64, and 65 of *The Organon,* which was followed by discussion.

Dr. Fincke read from his paper illustrations of the effects of drinking hot and cold water, wine, coffee, etc.

The Board of Censors reported in favor of several candidates for membership, and the Secretary was directed to cast the ballot for each name, by which they were declared elected.

The following is the list of names: Richard Hearn, Toronto, Canada; Overton F. McDonald, Toronto, Canada; Charles G. Wilson, Clarksville, Tenn.; W. J. Winn, Cambridgeport, Mass.; A. G. Allen, Philadelphia, Pa.; Clarence G. Selfridge, Port Townsend, State of Washington; Erastus E. Case, Hartford, Conn.; W. E. Ledyard, San Francisco, Cal.; B. Fincke, M. D., Brooklyn, N. Y.; Charles H. Oakes, Northboro', Mass.; George A. Taber, Richmond, Va.; J. M. Dutton, Boston,

---

* See page 382.

Mass.; W. P. Defriez, Brookline, Mass.; F. G. Davis, Quincy, Mass.; Jennie Medley, Philadelphia, Pa.

The Report of the Necrologist was received and adopted. It announced the death of Dr. Edward Bayard, New York,* Dr. David Wilson, of London,* Dr. L. S. Reynolds, of Brooklyn.†

SESSION OF JUNE 25TH.

*Morning.*—The President in the chair.

The Auditing Committee rendered their report, in which they paid a high compliment to the Treasurer, Dr. Clarence Willard Butler, of Montclair, N. J., for his successful efforts to free the Association from debt. They reported the accounts of the Secretary and Treasurer as correct.

Dr. W. A. Hawley, of Syracuse, was made Chairman of the Bureau of Homœopathics.

Dr. Stow, of Mexico, N. Y., was appointed Necrologist.

Dr. T. P. Wilson, of Ann Arbor, Mich., communicated a poem.

The Report of the Bureau of Obstetrics was made the order of the session.

Dr. James T. Kent read a paper upon the "Management of Displacements of the Uterus without Mechanical Support."

A long and interesting discussion followed.

Dr. J. B. Custis, of Washington, read an interesting paper entitled "The Hahnemannian Obstetrician." This was followed by another long and animated discussion upon the use of chloroform in labor; the use of forceps; the application of disinfectants, brandy, etc., in confinement.

Dr. Samuel A. Kimball, of Boston, then read a paper upon "Puerperal Fever."

This was succeeded by another discussion.

Dr. Wm. P. Wesselhœft, of Boston, then called attention to the need of a reliable post-graduate course where true Homœopathy could be certainly taught. He said: "Some of

---

* See HOMŒOPATHIC PHYSICIAN, December, 1889, pages 437, 441.

† See HOMŒOPATHIC PHYSICIAN, March, 1890, page 139.

the younger men in Boston have come to the older members of the homœopathic school, and have asked : 'Where can we learn this Homœopathy that you practice?' And no satisfactory answer can be given. The question has been asked us by men who were taking the medical course at Harvard. There has been some thought expended upon this subject. Now, if we could enlist two or three men in this work who would be willing to devote a portion of their time to giving instruction upon Homœopathy in their own offices to those who are earnest seekers after knowledge, much good might be accomplished. The fact that young men have asked the question 'Where can we go to hear something of Homœopathy?' is enough to incite us to an effort to answer this question. It is not only our duty to help these men, but to let it be known that there is a something which is Homœopathy and that it is still alive." Dr. Wesselhœft advocates a school for a post-graduate course in some one of the great cities; Boston would be a good place for such a school.

Dr. D. W. Clausen, of Philadelphia, wished to know if Dr. Wesselhœft was aware that such a course of lectures had been started in Philadelphia by Dr. Kent?

Dr. Wesselhœft answered that he had heard of such a course.

It was decided that the subject was so important that it should be made the special order of the evening session.

Dr. Butler then made report of the Committee upon the President's address, after which a discussion followed, and the Association adjourned until three o'clock P. M.

*Afternoon.*—Professor Edmund Carleton, of New York, read a paper upon tumors of the labia majora.

An interesting discussion was elicited, which followed many of the lines of thought of the morning.

A proposition for a Board of Honorable Seniors was then discussed, the idea being to advance to a special rank in the Association such members as had become distinguished by reason of their long and honorable career in the homœopathic practice, and to exempt them from the payment of dues to the Association. Such a Board would include veterans like Dr. P. P. Wells and Dr. E. A. Ballard.

Dr. Wesselhœft then read a paper, entitled "Dysmenorrhœa with Anemia."

Dr. Baylies read a paper proving the efficacy of Pulsatilla in preventing the malposition of the fœtus.

This was followed by a discussion which returned to the subject of Chloroform and other anæsthetics in labor. Dr. A. B. Campbell, of Brooklyn, opened up this discussion.

The report of the Bureau of Obstetrics being the next subject in order, papers were read by Drs. Hitchcock, Carleton, Brownell, and Dever.

Several papers from absent members were read by title. They were Drs. Hall, of Victoria, British Columbia; Dunlevy, Dillingham, and Myers, of New York.

Dr. Stow offered a resolution that all papers upon venereal diseases be referred to the Clinical Bureau. Carried.

*Evening.*—Dr. Bell addressed the meeting upon the subject of fumigation. The old school of medicine have been recommending and using fumes of Sulphur for disinfecting purposes. They now find that the anhydrous, or dry fumes, are of no use in disinfection. The Sulphur fumes must be moistened. He read an article from the *New York Medical Record*, showing that fumigation with dry Sulphur is a humbug.

Dr. H. C. Allen, of Chicago, editor of *The Medical Advance*, read a paper giving a proving of Kali-phosphoricum.

Dr. Case, being called upon, read a paper giving his own proving of Kali-phosphoricum.

Dr. W. L. Reed, of St. Louis, remarked that one of the provers of Kali-phos. had headache upon the right side of the head, which was relieved by gently rubbing the head with the hand, or stroking it. The pains were right-sided and supra-orbital.

Dr. Reed read a paper detailing a proving of Pyrogen. One of its symptoms was a terrible fetid taste in the mouth, as if an abscess had broken and discharged into the mouth. The taste was sweet and nasty.

Dr. Kent presented an interesting paper giving a proving of Cenchris contortrix, which provoked discussion.

Dr. Kimball read his paper upon an "Involuntary Proving of Secale." Discussion.

Dr. George H. Clark, of Germantown, Philadelphia, read a paper upon Asthenopia, with a very full eye repertory.

Dr. John V. Allen, of Frankford, Philadelphia, presented a complete repertory to nausea.

Dr. Wm. Jefferson Guernsey, of Philadelphia, read a paper, entitled "The Contrarieties of Ignatia."

Dr. H. C. Allen, of Chicago, moved that a vote of thanks be extended to Drs. Clark and Allen for their laborious repertories, the one upon the eyes, the other upon nausea. Carried.

Dr. J. B. G. Custis, of Washington, was appointed Chairman of the Bureau of Obstetrics.

Dr. Edward Rushmore, of Plainfield, N. J., was appointed Chairman of Bureau of Materia Medica.

SESSION OF JUNE 26TH.

*Morning.*—Bureau of Surgery was reopened.

Dr. James B. Bell, of Boston, related a case of laparotomy, with illustration.

In the discussion that followed, Dr. Bell said that surgery was the opprobrium of medicine. There should be no such thing as removal of tumors. There should be no tumors to remove. Surgery should be confined to the repairing of broken limbs, plastic operations and the like.

Dr. T. M. Dillingham, Chairman of the Bureau of Clinical Medicine, having at last arrived after being anxiously expected by the members during all the previous sessions, the report of his Bureau was considered.

Dr. J. W. Thomson read an interesting paper upon rheumatic gout, followed by discussion.

Dr. B. Fincke, of Brooklyn, read an interesting paper upon "Chronic Enlargement of the Testicle."

A vote of thanks was tendered to Dr. Fincke for his interesting paper.

An invitation was received from Dr. Rose, of Westerly, Rhode Island, for a visit to the town of Westerly, and an excursion in a specially chartered steamer. The invitation was declined owing to the shortness of time at disposal, and a unanimous vote of thanks tendered to Dr. Rose and to the citizens of Westerly.

Dr. Hoyne read a paper of clinical cases.

Dr. H. C. Allen read a paper upon a clinical case cured by Pyrogen. The Pyrogen tongue is clean, bright red, or fiery red, without elevated papillæ or cracks.

*Afternoon.*—Professor Edmund Carleton, of New York, was appointed Chairman of the Bureau of Surgery.

The Association then went into the election of officers.

The following officers were unanimously elected :

President, Dr. Clarence Willard Butler, of Montclair, N. J.

Vice-President, Dr. E. W. Sawyer, of Kokomo, Indiana.

Secretary, Dr. Samuel A. Kimball, of Boston.

Treasurer, Dr. Frank Powel, of Chester, Pa.

Corresponding Secretary, Dr. Wm. P. Wesselhœft, of Boston.

Chairman of the Board of Censors, Dr. Joseph A. Biegler.

Committee upon the place of next annual meeting was composed of Drs. Wesselhœft, Butler, Guernsey, Kent, and Hoyne.

It was understood that the committee would decide upon either Cresson Springs, Pa., or Atlantic City, N. J.

Dr. DeForrest Hunt resigned from membership on account of declining health.

Dr. Robert M. Fallon resigned on account of his entering the ministry.

Dr. Butler then proposed the following:

Amendment to Article III of the Constitution: "Except in case of Junior membership the applicant for which must only be a graduate of a recognized medical college."

Amendment to Section 3 of the By-laws :

"Application for Junior membership may be made by any physician six months in advance of a regular meeting and on the indorsement of three members of this Association, in good standing, and upon the recommendation of the Board of Censors; such physician may become a Junior member by a two-thirds vote of the members present. Such Junior members shall have the privilege of the floor for discussion of medical topics; shall be allowed to present such papers as are indorsed by the Board of Censors; shall be entitled to a copy of the Transactions of the Association, but shall not be entitled to vote, nor be eligible to office. At the expiration of three years of Junior membership, they may make application for full membership, subject to all conditions necessary for such application, or failing so to do their membership ceases."

On recommendation of the Board of Censors, any member of this Association, who, in their opinion, has rendered signal service to the cause of Hahnemannian Homœopathy or to the good and welfare of this Association, may be elected to the Board of Honorable Seniors, and as members of such Board shall retain all the rights and privileges of regular membership.

Amendment to Section 7 of By-laws:

"The annual dues of the regular members of this Association shall be five dollars. The annual dues of Junior members shall be two dollars, and all dues shall be payable in advance. Members of the Board of Honorable Seniors shall be released from the payment of annual dues."

Papers were presented by Drs. Whiting (read by Dr. H. C. Allen), Wesselhœft, Guernsey, Reed, Carleton, and Woods.

The paper of Dr. Reed, of St. Louis, was especially remarkable. After detailing a case of albumenuria, maltreated for nearly two years with massive doses of medicine and finally cured by Dr. Reed with Nat-mur., the doctor concluded with a peroration in which he indulged in vigorous invective against the mongrels or eclectics. He declared that the maltreatment of eclecticism was "enough to make the dead rise from the grave, the marble statue (of Hahnemann, at Leipsic) to step down from its pedestal," etc., evoking rounds of applause and cheers.

Dr. H. C. Allen, moved that in the Transactions the word "mongrel," wherever it occurs, be expunged.

Dr. Hawley inquired why?

Dr. Allen thought it offensive and exasperating and that we could catch more flies with sugar than with vinegar.

Dr. Hawley doubted that conclusion.

Dr. Thomson facetiously remarked that " we are not here to catch flies nor to hold a candle to the devil. We have a mission, which is to proclaim the truth regardless of results." The discussion then dropped.

Dr. Baylies was appointed Chairman of Bureau of Clinical Medicine.

### SESSION OF JUNE 27TH.

*Morning.*—The Association proceeded to the consideration of the publication of the Transactions. Dr. Kent moved that the proceedings be given to *The Medical Advance*, to publish upon the same terms as last year.

Dr. James, Editor of THE HOMŒOPATHIC PHYSICIAN, made a bid.

Dr. Allen, of *The Medical Advance*, offered the very same terms as Dr. James.

Dr. Hitchcock, of *The Journal of Homœopathics*, made a bid.

Discussion followed, and it was finally decided to refer the whole matter to the Executive Committee with power to give the work to the best bidder.

The report of the Bureau of Clinical Medicine was now reopened.

Dr. Kimball read a paper upon " Epilepsy," which was followed by a discussion, in which Dr. Carleton declared that he had not listened to any paper that had given him so much pleasure as the one just read by Dr. Kimball.

Papers were then read by Dr. Rushmore, Dr. Farley, Dr. Sawyer, and Dr. Dillingham.

A vote of thanks was tendered Dr. Dillingham for his admirable report of cases cured.

Dr. Butler preferred charges against Dr. Gentry on account of an article in *The American Homœopathist* for June, written by Dr. Gentry, in which he advocated the use of large doses of Quinine to antidote the malarial poison. The charges were referred to the Board of Censors.

Dr. Thomson offered a copy of a letter written to the *Sun* of

New York City, protesting against some communications which had appeared denouncing Homœopathy. The *Sun* refused to publish the defense, and so Dr. Thomson presented it to the Association. It was accepted.

The Committee upon place of next annual meeting reported in favor of Cresson Springs, or else Atlantic City. The report was accepted and the Committee discharged. It was then referred to the Executive Committee.

A vote of thanks was tendered to Dr. Biegler for his able and just rulings as President.

A vote of thanks was then given the proprietor of the Ocean House, where the meeting was convened, after which the Association adjourned until next year.

